# The Effect of Dysglycaemia on Changes in Pulmonary and Aerobic Function in Cystic Fibrosis

**DOI:** 10.3389/fphys.2022.834664

**Published:** 2022-03-30

**Authors:** Owen W. Tomlinson, Anna L. E. Stoate, Lee Dobson, Craig A. Williams

**Affiliations:** ^1^Children’s Health and Exercise Research Centre, Sport and Health Sciences, University of Exeter, Exeter, United Kingdom; ^2^Royal Devon and Exeter NHS Foundation Trust Hospital, Exeter, United Kingdom

**Keywords:** cystic fibrosis related diabetes, cardiorespiratory fitness, oxygen uptake, longitudinal data, pulmonary disease

## Abstract

Cross-sectional studies have reported lower pulmonary and aerobic function during exercise in people with cystic fibrosis-related diabetes (CFRD) compared to non-CFRD counterparts. However, this association has yet to be longitudinally investigated. Therefore, this study examines these differences over time between people with cystic fibrosis (CF) of differing glycaemic status. Annual review data, including cardiopulmonary exercise tests and pulmonary function tests, were retrospectively analysed at baseline (T0, *n* = 82) and at a one-year follow-up (T1, *n* = 54). Data was analysed in three groups: normal glucose tolerance (NGT), impaired glucose tolerance (IGT), and CFRD. Further analyses were undertaken, with a dichotomous split of NGT and a combined IGT/CFRD group. At baseline, a significant reduction in the majority of variables, including forced expiratory volume in one second (FEV_1_) and maximal oxygen uptake (VO_2max_), was observed in the CFRD (*n* = 19) group compared to NGT (*n* = 58). At follow-up, no significant differences were observed, and no interaction effect between CFRD status and time was identified. FEV_1_ and VO_2max_ presented with varying directions and magnitudes of change within patients. In summary, patients with CFRD have a reduced aerobic and pulmonary function compared to non-CFRD counterparts, although such changes disappeared at follow up. Varying responses for FEV_1_ and VO_2max_ highlight the need to consider both variables as independent markers of function in CF.

## Introduction

Cystic fibrosis (CF), the most common autosomal recessive condition in the Caucasian population, affecting ∼10,500 people in the United Kingdom (UK) (Cystic Fibrosis Trust, 2019). Due to increased life expectancy, non-respiratory co-morbidities are becoming more significant contributors to ill-health and prognosis (Hebestreit et al., 2019; Keogh et al., 2019).

Estimates show that 85% of people with CF have a degree of pancreatic insufficiency (PI) (Singh and Schwarzenberg, 2017), and in 2018, 30% of people over 10 years of age were undergoing treatment for CF-related diabetes (CFRD) in the UK (Cystic Fibrosis Trust, 2019). It is believed incidence of CFRD rises with age, with increasing reported prevalence from 11 to 24% over 5 years (Lanng et al., 1995). Given that the pancreas is one of the earliest affected organs in CF (Gibson-Corley et al., 2016) and insufficiency is becoming a major determinant of morbidity and mortality (Causer et al., 2020), the number of people being screened for CFRD has increased from 42% in 2004 to 81% in 2018 (Ukcf Database, 2006; Cystic Fibrosis Trust, 2019). As prevention of CFRD is cited as a research priority within the CF community (Rowbotham et al., 2018), there is a necessity for research that explore factors associated with CFRD.

Several studies indicate that glucose intolerance is associated with poor clinical function (Ziegler et al., 2011; Foster et al., 2018; Causer et al., 2020), although some report contradicting results in relation to CFRD and lung function. For example, forced expired volume in one second (FEV_1_) and forced vital capacity (FVC) have been reported to be reduced in CFRD (Koch et al., 2001; Causer et al., 2020), but a lack of difference (relative to those with normal glucose tolerance, NGT) is reported elsewhere (Ziegler et al., 2011; Foster et al., 2018). Given the variability in the relationship between CFRD and FEV_1_, alternative factors must be investigated to create a detailed and holistic clinical profile for patients.

With cardiopulmonary exercise testing (CPET) acknowledged as the gold-standard assessment for determining maximal oxygen uptake (Hebestreit et al., 2015), it has been established that higher levels of aerobic fitness (represented by peak/maximal oxygen uptake, VO_2peak/max_) are important in CF. Higher levels of aerobic fitness are associated with reduced risk of hospitalisation and better prognosis (Pérez et al., 2014; Hebestreit et al., 2019). To date, minimal research exists on how CFRD affects aerobic fitness, although a single cross-sectional study established that people with CFRD have reduced aerobic fitness compared to non-CFRD counterparts (Causer et al., 2020). When considering longitudinal designs, research has assessed temporal interactions between glycaemic status, aerobic fitness, and physical activity, finding that CFRD negatively impacts changes in FEV_1_ (Schneiderman et al., 2014). However, this latter study did not explicitly identify the association between glycaemic status and aerobic fitness over time. Therefore, this lack of data provides the rationale for the present analysis.

This study sought to investigate differences in pulmonary and aerobic function in people with CF of differing glycaemic status, generating novel longitudinal data on the relationship between these factors, whilst providing robust replication of previous cross-sectional analyses (Causer et al., 2020).

## Materials and Methods

### Study Design and Ethics

A retrospective analysis of clinical data from the Royal Devon and Exeter Cystic Fibrosis Centre, collected between 2015 and 2018, was performed. As per national guidelines (National Institute for Health and Care Excellence, 2017), data collected at annual review includes pulmonary function testing, nutritional and diabetic review, and exercise testing. This data is recorded *via* a standardised *proforma* (Cystic Fibrosis Trust, 2021) for use by the national registry, with study data collated using this platform.

As this study analysed retrospective, routinely collated data, and was anonymised prior to analysis, full ethical review and patient consent was not required. Approval for use of anonymised data was obtained from the Health Research Authority (IRAS 238996).

### Participants and Timeline

All patients included in this assessment had confirmed diagnosis of CF based on clinical features, elevated sweat chloride (>60 mmol L^–1^), and genotyping where possible. The CFTR2 database (Cystic Fibrosis Foundation, 2011) assisted with genotype classification, with Class I/II mutations regarded as “severe.”

Data were analysed from two time-points: baseline (T0) and at one-year follow-up (T1). Each patient’s baseline measure was not necessarily in the same year as one another, but represented the first year they performed CPET at annual review. Regardless of T0 date, T1 measures were one-year later for all patients.

### Anthropometry and Pulmonary Function

Body mass and stature were measured to the nearest 0.1 kg and 0.01 m, respectively, with body mass index subsequently calculated. Pulmonary function (FEV_1_ and FVC) was assessed *via* flow-volume loop spirometry, with results recorded as absolute values and as percent of predicted (%_Pred_), using normative values from the Global Lung Initiative (Quanjer et al., 2012).

### Glycaemic Status

Through oral glucose tolerance testing and continuous glucose monitoring, both of which are utilised in CF management (Cystic Fibrosis Trust, 2004), participants were categorised into NGT (<7.8 mmol L^–1^), impaired glucose tolerance (IGT; 7.8–11.0 mmol L^–1^), and CFRD (≥11.1 mmol L^–1^). These boundaries are in line with existing guidelines (American Diabetes Association, 1997) and have been adopted by previous CFRD studies to assess exercise (Causer et al., 2020). Glycaemic status data was obtained from annual review *proforma* (Cystic Fibrosis Trust, 2021).

### Cardiopulmonary Exercise Testing

Cardiopulmonary exercise testing was performed *via* cycle ergometry (Lode Excalibur; Lode, Groningen, Netherlands). Breath-by-breath pulmonary gas exchange using a metabolic cart (Metalyzer II; Cortex Biophysik, Leipzig, Germany) determined VO_2peak_ which was subsequently confirmed as a maximal VO_2_ (VO_2max_) by supra-maximal verification (S_max_) at 110% peak power output (PPO) of that achieved in an initial ramp-incremental test (10–30 W min^–1^) to volitional exhaustion; a process validated in both adults and children with CF (Saynor et al., 2013; Causer et al., 2018). If VO_2max_ could not be verified *via* S_max_ testing, secondary criteria were utilised to ascertain maximal efforts (Radtke et al., 2019).

All CPET derived variables were interpolated to 10 s averages, with highest values taken as the peak was achieved. A slope of ventilatory equivalents of CO_2_ (V_*E*_/VCO_2_) was established from the start of the ramp test to the respiratory compensation point. The gas exchange threshold (GET) was calculated using the V-slope method (Beaver et al., 1986), and corroborated using ventilatory equivalents of CO_2_ and O_2_.

### Data Analysis

Firstly, for cross-sectional analyses at baseline and follow-up, one-way analyses of variance (ANOVA) with *post hoc* Bonferroni-corrected independent-sample *t*-tests identified main effects of a group upon variables. In addition, with specific relation to VO_2max_ (mL kg^–1^ min^–1^) and analysis of covariance (ANCOVA), controlling for FEV_1_ (%_Pred_) was undertaken at both time-points to identify effect of group, independent of lung function. Pearson’s correlation coefficients established relationships between FEV_1_ and VO_2max_.

Secondly, to identify changes over time, a mixed-model ANOVA determined interaction effects between groups and time for FEV_1_ and VO_2max_, from T0 to T1. This mixed-model ANOVA used dichotomous groups, whereby IGT patients were included within the CFRD group (due to having a degree of PI) to counter reduced statistical power due to loss of follow-up data. *Post hoc* Bonferroni-corrected paired-sample *t*-tests highlighted significant differences between variables at differing time-points. Additionally, mixed-model ANCOVAs were specifically performed for VO_2max_ (mL kg^–1^ min^–1^), controlling for FEV_1_ (%_Pred_) and age (years). Chi-square tests and logistic regression identified associations between direction of change over time and glycaemic status, along with probability of any increase/decrease in either FEV_1_ or VO_2max_. Pearson’s correlation coefficients established relationships between changes in both FEV_1_ and VO_2max_.

Data are expressed as means (± standard deviation). Effect sizes (*ES*) were expressed using thresholds of Cohen’s *d* describe differences between groups (small 0.2 < 0.5, medium 0.5 < 0.8, large ≥ 0.8) and correlation coefficients (small 0.1 < 0.3, medium 0.3 < 0.5, large ≥ 0.5) (Cohen, 1992). Analyses were performed using SPSS v.26 (IBM; Armonk, NY, United States), with *p* < 0.05 indicating statistical significance.

## Results

### Patient Inclusion and Follow-Up

**FIGURE 1:**
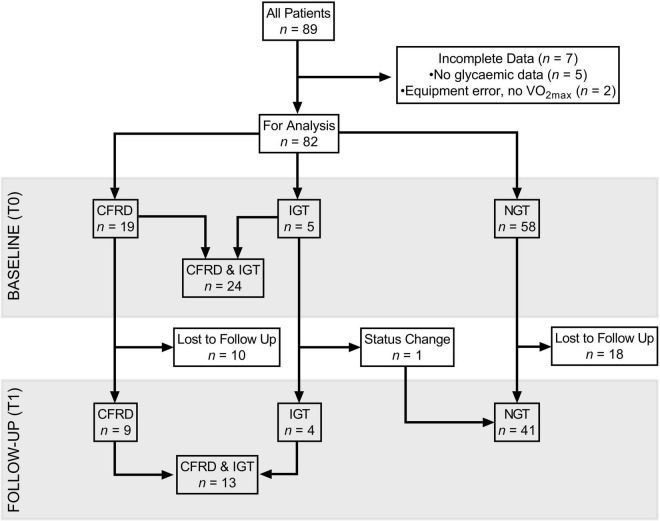
Flow diagram, characterising follow-up and drop-out of participants, split by glycaemic status in three groups (CFRD, IGT, NGT), and when considered dichotomously (IGT/CFRD, NGT) at baseline and one-year follow-up. CFRD: cystic fibrosis related diabetes; IGT: impaired glucose tolerance; NGT: normal glucose tolerance; T0: baseline measurement; T1: 1 year follow-up; VO_2max_: maximal oxygen uptake.

Records identified *n* = 89 patients who had undergone CPET within the specified timeframe and were eligible for inclusion. However, due to incomplete clinical datasets, *n* = 82 were included in analyses at T0 [paediatric (<18 years), *n* = 31]. Of the *n* = 7 excluded at T0, *n* = 2 were removed due to equipment errors during CPET, resulting in failure to obtain accurate VO_2max_ (primary exercise variable), and *n* = 5 due to lack of information on glycaemic status (Figure 1). The characteristics of included patients at T0 and T1 are shown in Tables 1, 2, respectively.

**TABLE 1:** Participant characteristics including anthropometry, pulmonary function, and exercise-based outcomes at baseline (T0).

	NGT (*n* = 58)	IGT (*n* = 5)	CFRD (*n* = 19)	*P*-value
**Participants**				
Males (%)	64	60	42	–
Age (y)	25.8 ± 14.5	22.2 ± 8.0	27.0 ± 10.5	0.78
ΔF508 Status* (*n*)	18/35/5	4/1/0	7/10/2	–
Pancreatic Insufficient (*n*)	34	5	19	–
**Anthropometry**				
Stature (m)	1.65 ± 0.13	1.66 ± 0.12	1.64 ± 0.8	0.93
Body mass (kg)	65.3 ± 17.9	60.0 ± 11.3	59.5 ± 11.4	0.36
BMI (kg m^–2^)	23.42 ± 4.15	21.87 ± 3.21	21.94 ± 2.80	0.27
**Spirometry**				
FVC (L)	3.76 ± 1.19	3.45 ± 0.95	3.06 ± 0.85	0.06
FVC (%_Pred_)	90.8 ± 13.7	81.4 ± 14.4	76.1 ± 19.9	**<0.01*^a^***
FEV_1_ (L)	2.83 ± 1.03	2.62 ± 0.73	1.97 ± 0.83	**0.01*^a^***
FEV_1_ (%_Pred_)	81.6 ± 18.9	72.8 ± 12.6	58.2 ± 23.9	**<0.01*^a^***
**Exercise**				
PPO (W)	172 ± 63^†^	149 ± 61	126 ± 52	**0.02*^a^***
PPO (W kg^–1^)	2.68 ± 0.75^†^	2.41 ± 0.56	2.11 ± 0.74	**0.02*^a^***
VO_2max_ (L min^–1^)	1.88 ± 0.65	1.44 ± 0.44	1.41 ± 0.44	**<0.01*^a^***
VO_2max_ (mL kg^–1^ min^–1^)	29.44 ± 8.30	23.66 ± 2.75	23.69 ± 5.93	**0.01*^a^***
HR_peak_ (beats min^–1^)	175 ± 18^†^	181 ± 11^†^	161 ± 13^†^	**0.03*^a^***
GET (mL kg^–1^ min^–1^)	17.24 ± 4.81^†^	14.99 ± 2.38	13.77 ± 3.36^†^	**0.02*^a^***
GET (%VO_2max_)	59.3 ± 10.7^†^	63.3 ± 7.3	57.4 ± 10.5^†^	0.54
V_Epeak_ (L min^–1^)	92.47 ± 37.24	94.38 ± 30.13	68.62 ± 22.54	**0.03*^a^***
RER	1.58 ± 0.34	1.87 ± 0.23	1.49 ± 0.27	0.06
V_*E*_/VCO_2_	35.4 ± 5.1	44.5 ± 5.1	34.2 ± 4.7	**<0.01*^b^***

Data are expressed as means ± standard deviation. *ΔF508 status provided as homozygous/heterozygous/none. Text in bold demonstrates a significant main effect of group, obtained from one-way ANOVA (p < 0.05), driven by significant post hoc (p < 0.05) between normal glucose tolerance (NGT) and cystic fibrosis-related diabetes (CFRD) (a) or NGT and impaired glucose tolerance (IGT) (b). Incomplete data due to aforementioned reasons at T0 (†): peak power output (PPO; NGT, n = 56); peak heartrate (HR_peak_; NGT, n = 41; IGT, n = 4; CFRD, n = 13); gas exchange threshold (GET; NGT, n = 57; CFRD, n = 16). BMI: body mass index, FVC: forced vital capacity, FEV_1_: forced expiratory volume in 1 s, VO_2max_: maximal oxygen uptake, HR_peak_: peak heart rate, GET: gas exchange threshold, PPO: peak power output, RER: respiratory exchange ratio, V_Epeak_: peak minute ventilation, V_E_/VCO_2_: ventilatory equivalent for carbon dioxide, NGT: Normal glucose tolerance, IGT: Impaired glucose tolerance, CFRD: cystic fibrosis related diabetes.

**TABLE 2:** Participant characteristics including anthropometry, pulmonary function, and exercise -based outcomes at one-year follow-up (T1).

	NGT (*n* = 41)	IGT (*n* = 4)	CFRD (*n* = 9)	*P*-value
**Participants**				
Males (%)	71	50	33	–
Age (y)	26.1 ± 12.7	24.7 ± 8.4	24.8 ± 10.4	0.95
ΔF508 Status* (*n*)	17/19/5	3/1/0	4/4/1	–
Pancreatic Insufficient (*n*)	25	4	8	–
**Anthropometry**				
Stature (m)	1.68 ± 0.12	1.61 ± 0.13	1.63 ± 0.09	0.37
Body mass (kg)	67.3 ± 16.2	59.4 ± 13.9	62.1 ± 15.6	0.48
BMI (kg m^–2^)	23.48 ± 3.94	22.46 ± 2.05	22.89 ± 3.30	0.82
**Spirometry**				
FVC (L)	3.94 ± 1.11	3.68 ± 1.32	3.28 ± 0.81	0.25
FVC (%_Pred_)	88.8 ± 12.0	91.0 ± 18.9	83.9 ± 20.1	0.58
FEV_1_ (L)	2.85 ± 0.99	2.71 ± 0.73	2.17 ± 0.93	0.17
FEV_1_ (%_Pred_)	77.2 ± 18.6	79.1 ± 6.3	63.9 ± 24.4	0.17
**Exercise**				
PPO (W)	196 ± 68	154 ± 71	154 ± 59	0.15
PPO (W kg^–1^)	2.92 ± 0.75	2.49 ± 0.63	2.45 ± 0.61	0.15
VO_2max_ (L min^–1^)	2.02 ± 0.63	1.56 ± 0.71	1.58 ± 0.43	0.08
VO_2max_ (mL kg^–1^ min^–1^)	30.38 ± 7.48	25.24 ± 5.85	25.55 ± 3.68	0.09
HR_peak_ (beats min^–1^)	176 ± 26^†^	181^†^	181 ± 17^†^	0.90
GET (mL kg^–1^ min^–1^)	17.46 ± 4.89	13.29 ± 2.09	14.66 ± 4.01^†^	0.10
GET (%VO_2max_)	58.1 ± 10.5	53.8 ± 9.6	57.4 ± 10.6^†^	0.73
V_Epeak_ (L min-^1^)	100.50 ± 34.97	92.24 ± 34.40	83.52 ± 27.40	0.39
RER	1.70 ± 0.38	1.91 ± 0.33	1.78 ± 0.23	0.48
V_*E*_/VCO_2_	35.3 ± 5.6	37.8 ± 3.9	35.6 ± 4.9	0.68

Data are expressed as means ± standard deviation. *ΔF508 status provided as homozygous/heterozygous/none. P-values obtained from one-way ANOVA. Incomplete data due to aforementioned reasons at T1 (†): HR_peak_ (NGT, n = 22; IGT, n = 1; CFRD, n = 6); GET (CFRD, n = 8). BMI: body mass index, FVC: forced vital capacity, FEV_1_: forced expiratory volume in 1 s, VO_2max_: maximal oxygen uptake, HR_peak_: peak heart rate, GET: gas exchange threshold, PPO: peak power output, RER: respiratory exchange ratio, V_Epeak_: peak minute ventilation, V_E_/VCO_2_: ventilatory equivalent for carbon dioxide, NGT: Normal glucose tolerance, IGT: Impaired glucose tolerance, CFRD: cystic fibrosis related diabetes.

At T1, *n* = 54 (paediatric *n* = 17) patients were included. Of those lost to follow-up (i.e., no CPET at T1), 35% in the NGT and 82% in the IGT/CFRD group were identified as having severe mutations. A breakdown of genotypes is provided in Supplementary Material.

### Validity of Cardiopulmonary Exercise Testing

At T0, 40% of participants did not have VO_2max_ verified *via* S_max_ tests (*n* = 11 did not perform S_max_, *n* = 22 not verified despite undergoing S_max_), although all satisfied secondary criteria were used to verify maximal efforts. At T1, 31% did not have a verified VO_2max_ (*n* = 2 did not perform S_max_, *n* = 15 not verified), although all patients satisfied secondary criteria. Consequently, as all participants satisfied criteria for maximal efforts, the term “VO_2max_” is used herein.

Moreover, within Tables 1, 2, a number of variables are unavailable due to equipment malfunctions during CPET (HR_max_), accidental omission of data recording during testing (PPO), and non-detection of GET. However, despite some missing data, patients have been continued forward for analyses as they still possessed data pertaining to VO_2max_ – the primary exercise variable of interest.

Finally, analyses revealed that allometric scaling (Armstrong and Welsman, 1994) was not required for VO_2max_ as no significant correlation was identified between body mass and ratio-scaled VO_2max_ (*r* = −0.19, *p* = 0.09).

### Baseline Data

One-way ANOVA showed a significant main effect of group in the majority of pulmonary and exercise-related variables, including FEV_1_ and VO_2max_ (Table 1). Predominantly, these significant results were driven by differences between NGT and CFRD, apart from V_*E*_/VCO_2_ – driven by differences between NGT and IGT. When ANCOVA was undertaken for VO_2max_ (controlling for FEV_1_), the main effect of group disappeared (*p* = 0.25).

**FIGURE 2:**
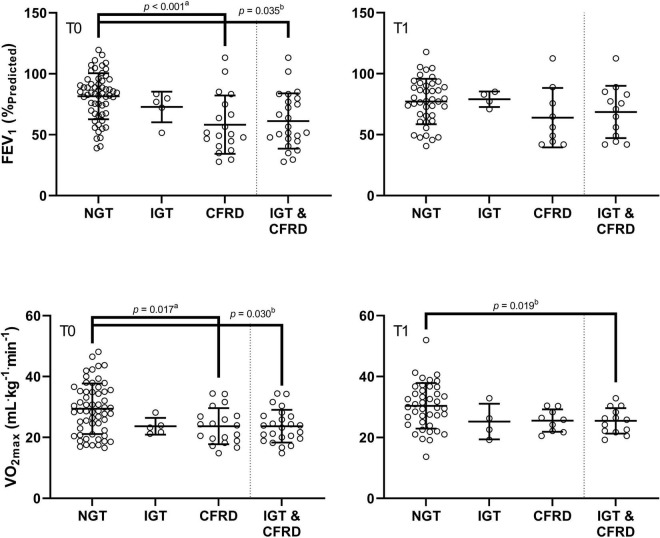
Comparison of FEV_1_ (%_Pred_) and VO_2max_ in groups of differing glycaemic status at baseline (T0) and at one-year follow up (T1). CFRD: cystic fibrosis related diabetes; IGT: impaired glucose tolerance; NGT: normal glucose tolerance. FEV_1_: forced expiratory volume in 1 s as a percentage of predicted; VO_2max_: maximal oxygen uptake. *P*-values are derived from *post hoc* tests following one-way ANOVA (a), and repeated measures ANOVA (b).

In assessing combined groups, *post hoc* analyses from the mixed design ANOVA revealed significantly higher FEV_1_ within the NGT group relative to the combined IGT/CFRD group at baseline (80.90 ± 20.50 vs. 67.11 ± 20.41%_Pred_, *p* = 0.035, *ES* = 0.67, Figure 2). Furthermore, a significantly higher VO_2max_ was identified in the NGT group relative to the combined IGT/CFRD group (30.37 ± 7.80 vs. 25.38 ± 4.94 ml kg^–1^ min^–1^, *p* = 0.030, *ES* = 0.69, Figure 2). However, when FEV_1_ was controlled for in a mixed-model ANCOVA, this became non-significant (*p* = 0.14), whereas controlling for age ensures that significance between groups is maintained (*p* = 0.013).

The correlation between FEV_1_ and VO_2max_ was medium and statistically significant for the whole group (*r* = 0.44, *p* < 0.001), and the NGT group (*r* = 0.38, *p* = 0.003). A medium, non-significant coefficient was present in the IGT/CFRD group (*r* = 0.33, *p* = 0.12).

### One-Year Follow-Up

One-way ANOVA showed no main effect of group for any variable (Table 2). Moreover, for VO_2max_, one-way ANCOVA identified no significant effect of group (*p* = 0.17).

When assessing data using combined groups, no significant difference was evident for FEV_1_, although a medium effect size remained (NGT = 78.47 ± 18.50, IGT/CFRD = 67.20 ± 21.24%_Pred_, *p* = 0.06, *ES* = 0.59). A higher VO_2max_ was observed in the NGT group relative to the combined IGT/CFRD group (30.21 ± 7.31 vs. 25.20 ± 4.14 ml kg^–1^ min^–1^, *p* = 0.019, *ES* = 0.75, Figure 2), although this significance disappeared when controlling for FEV_1_ in ANCOVA models (*p* = 0.13) but remained when age was controlled for (*p* = 0.005).

The correlation between FEV_1_ and VO_2max_ was medium and statistically significant for the whole group (*r* = 0.44, *p* = 0.001) and the NGT group (*r* = 0.48, *p* = 0.001), but not the IGT/CFRD group (*r* = 0.12, *p* = 0.59).

### Longitudinal Changes

One patient (adolescent male) changed from IGT to NGT between T0 and T1 (Figure 1). All other patients remained stable with regards to glycaemic status.

No significant difference was identified for FEV_1_ over time in the combined IGT/CFRD group (67.11 ± 20.41 vs. 67.20 ± 21.24%_Pred_, *p* = 0.97, *ES* = 0.00), whereas a near-significant difference was identified within the NGT group (80.90 ± 20.50 vs. 78.47 ± 18.50%_Pred_, *p* = 0.051, *ES* = 0.12). No significant differences were observed with regards to VO_2max_ in either the NGT (30.37 ± 7.80 vs. 30.21 ± 7.31 ml kg^–1^ min^–1^, *p* = 0.83, *ES* = 0.02) or combined IGT/CFRD group (25.38 ± 4.94 vs. 25.20 ± 4.14 ml kg^–1^ min^–1^, *p* = 0.90, *ES* = 0.04).

Within the NGT group, FEV_1_ and VO_2max_ increased in 35 and 50% of patients, respectively. Within the IGT/CFRD group, FEV_1_ and VO_2max_ similarly increased by 36 and 50%, respectively. Association between glycaemic status and direction of change resulted in non-significant Chi-square tests for both FEV_1_ (χ^2^ < 0.01, *p* = 0.96) and VO_2max_ (χ^2^ < 0.01, *p* = 1.00) and non-significant logistic regressions for both FEV_1_ (β = 1.03, *p* = 0.96) and VO_2max_ (β = 1.00, *p* = 1.00).

**FIGURE 3:**
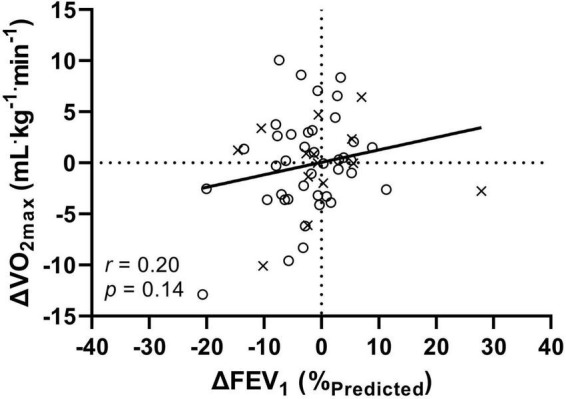
The relationship between the annual change (Δ) in FEV_1_ and VO_2max_ for 54 patients with CF. Crosses (×) indicate combined IGT/CFRD group, circles (°) indicate NGT group. FEV_1_: forced expiratory volume in 1 s; VO_2max_: maximal oxygen uptake. All data is presented as T1-T0 (i.e., a negative number indicates a decrease in function over the course of 1 year).

The correlation between the change in FEV_1_ and VO_2max_ was small but not statistically significant at the whole group level (*r* = 0.20, *p* = 0.14, Figure 3), within the NGT (*r* = 0.27, *p* = 0.09) or IGT/CFRD groups (*r* = 0.07, *p* = 0.80).

## Discussion

This is the first study to longitudinally examine how dysglycaemia impacts upon pulmonary and aerobic function in patients with CF. Three major findings are reported: (1) at baseline, patients with CFRD had statistically lower function in most pulmonary and aerobic function measures; (2) at follow-up, no statistically significant differences were found between groups for any measure; although when dichotomously grouped, statistically lower values were found for VO_2max_ within the CFRD/IGT group; and (3) VO_2max_ and FEV_1_ presented with contrasting magnitude and direction of change over time.

Previous studies have established associations between poorer glycaemic status and reduced pulmonary and aerobic function (Lanng et al., 1995; Koch et al., 2001; Foster et al., 2018), although this finding is inconsistent (Ziegler et al., 2011) and alternative indices of health status should be considered (i.e., VO_2max_). Previous cross-sectional exercise-oriented research from Causer et al. (2020) found no significant differences between groups of differing glycaemic status for parameters of PPO, HR_peak_, V_Epeak_, and V_*E*_/VCO_2_, conflicting with our findings. However, VO_2max_ within the work of Causer et al. (2020) was higher in the NGT group than the present study, and V_*E*_/VCO_2_ – which is linked to decreased pulmonary perfusion (Arena et al., 2004) – was also higher in the present work. Therefore, reduced VO_2max_ in the current group and a difference in sample sizes between studies likely explains the observed differences. Additionally, Causer et al. (2020) identified lower VO_2max_ in those with PI compared to their non-CFRD counterparts, which is in accordance with the present study.

The novelty of the present study is that it provides longitudinal data on both pulmonary and exercise function, with no significant differences found between groups at follow-up. However, when dichotomously split to create a combined IGT/CFRD group, a non-significant (albeit with medium *ES*) difference was observed for FEV_1_, and VO_2max_ was significantly different between groups. Moreover, the further use of ANCOVAs within these analyses indicate that age does not account for the difference in fitness between groups – a useful factor to consider as children will typically have yet to develop CFRD (unlike adult counterparts) and may bias the composition of a NGT groups. However, in contrast, FEV_1_ was shown to likely account for differences in fitness between groups. Therefore, further evidencing how disease progression impacts upon multiple parameters and organ systems. In addition, when comparing change over time, whilst no significant group-level differences were observed, approximately 65% of patients presented with declines in FEV_1_, whereas 50% declined in VO_2max_. The direction and magnitude of individual changes were highly variable (Figure 3), whereby such variances may reveal systematic exercise-oriented stability in CF, warranting investigation into whether cardiovascular and/or musculoskeletal function compensates for changing pulmonary function in order to maintain aerobic fitness.

The link between impaired glycaemic status and poorer pulmonary and aerobic capacity has a number of possible explanations. Dysglycaemia triggers oxidative stress and inflammation (Lanng et al., 1995) which can, in turn, induce microvascular dysfunction in the lungs (Totani et al., 2017). Furthermore, diabetes mellitus has an established effect on the immune system in addition to increased levels of glucose in the airways, increasing bacterial proliferation, and placing patients with endocrine PI at greater risk of pulmonary infection, thereby decreasing function. Along with the infection risk, insulin is a potent anabolic hormone, with deficiency promoting catabolism and malnutrition and impairing lung function *via* reduced respiratory muscle mass (Gibson-Corley et al., 2016). Thus, given suggested mechanisms, it is therefore unsurprising that baseline data indicated that patients with CFRD had poorer performance compared to non-CFRD counterparts. Whilst results were expected at baseline in accordance with previous work (Causer et al., 2020), it is surprising to see that differences were not maintained at one-year follow up.

One potential explanation for the lack of significant results at one-year follow up is the loss of patients (*n* = 82 at T0 vs. *n* = 54 at T1). This will impact upon statistical power. For example, upon the medium effect size between groups for FEV_1_ at T1 that failed to reach statistical significance (*ES* = 0.59, *p* = 0.06) or the non-significant difference between time-points for FEV_1_ (*p* = 0.051). Replication of the present study with an increased sample would likely result in statistical significance for these effects.

Moreover, those within the NGT group tended toward milder phenotypes than those with IGT/CFRD, whereas at both time-points, the IGT/CFRD group had a greater proportion of those with severe mutations (Cystic Fibrosis Foundation, 2011) (Supplementary Material). A larger percentage of those with severe mutations, and potentially with more severe disease, were lost to follow-up within the IGT/CFRD group. This is supported by the fact that patients in the IGT/CFRD group received more antibiotics than the NGT group over the year (Supplementary Material) and may be indicative of disease progression in this group. Subsequently, those that remained at T1 in the IGT/CFRD group may have had a better pulmonary and aerobic capacity (relative to “lost” IGT/CFRD counterparts), and therefore performed with greater similarity to those in the NGT group. Whilst every endeavour is made to perform annual exercise tests on each patient, this may not always happen. In addition, given that pulmonary function is a predictor of whether patients will undergo annualised CPET (Tomlinson et al., 2020), it is feasible that disease progression has directly affected follow-up results being obtained in this cohort.

There are a number of strengths to this study, including a larger sample size relative to previous studies (Causer et al., 2020) (thus providing increased external validity) and use of gold-standard CPET to ascertain VO_2max_ (Hebestreit et al., 2015). Moreover, analyses were performed on routinely collected data from a single CF centre. Therefore, this group was unlikely to have included heterogeneous treatment regimens that may introduce bias, along with treatments that were occurring prior to the widespread introduction of CFTR modulator therapies, which may impact results. Whilst a strength of the study includes analysis of both children and adults, maturational status should be acknowledged as a potential confounding issue. Insulin resistance increases during puberty, potentially altering CFRD status, although the decline in sensitivity is accompanied by compensatory insulin secretion and recovery after completion of puberty (Kelsey and Zeitler, 2016). This is anecdotally observed within this study, as the only participant to change their glycaemic status was an adolescent male.

In summary, this study found reduced pulmonary function and aerobic fitness in people with CF who also exhibited impaired glycaemic status at a baseline observation, although no significant differences were observed at follow-up. Furthermore, there appeared to be stability in exercise function relative to pulmonary function, thus furthering the evidence for considering VO_2max_ as an independent clinical maker in the assessment and prospective management of CF.

## Data Availability Statement

The raw data supporting the conclusions of this article will be made available by the authors, without undue reservation. Please contact the corresponding author, CW.

## Ethics Statement

Ethical review and approval was not required for the study on human participants in accordance with the local legislation and institutional requirements. Written informed consent from the participants’ legal guardian/next of kin was not required to participate in this study in accordance with the national legislation and the institutional requirements.

## Author Contributions

OT: conceptualisation, data acquisition, data analysis, data interpretation, manuscript review, and editing. AS: data analysis, data interpretation, manuscript drafting, review, and editing. LD: data interpretation, manuscript review, and editing. CW: conceptualisation, data analysis, data interpretation, manuscript review, and editing. All authors have approved final manuscript for publication and agreed to be accountable for all aspects of the work.

## Conflict of Interest

The authors declare that the research was conducted in the absence of any commercial or financial relationships that could be construed as a potential conflict of interest.

## Publisher’s Note

All claims expressed in this article are solely those of the authors and do not necessarily represent those of their affiliated organizations, or those of the publisher, the editors and the reviewers. Any product that may be evaluated in this article, or claim that may be made by its manufacturer, is not guaranteed or endorsed by the publisher.

## Abbreviations

ANCOVA: analysis of covariance
ANOVA: analysis of variance
BMI: body mass index
CF: cystic fibrosis
CFRD: cystic fibrosis related diabetes
CPET: cardiopulmonary exercise testing
ES: effect size
FEV_1_: forced expiratory volume in one second
FVC: forced vital capacity
GET: gas exchange threshold
HR_max_: maximal heart rate
IGT: impaired glucose tolerance
NGT: normal glucose tolerance
PI: pancreatic insufficiency
PPO: peak power output
RER: respiratory exchange ratio
S_max_: supramaximal verification testing
T0: baseline data
T1: one-year follow up data
UK: United Kingdom
V_Epeak_: peak minute ventilation
V_*E*_/VCO_2_: ventilatory equivalent of carbon dioxide
VO_2max_: maximal oxygen uptake
VO_2peak_: peak oxygen uptake.
