# Supplementary material for: The Effect of Dysglycaemia on Changes in Pulmonary and Aerobic Function in Cystic Fibrosis
Source: Front Physiol. 2022 Mar 30;13:834664. doi: 10.3389/fphys.2022.834664 (PMC9005891; doi:10.3389/fphys.2022.834664)
Supplement: Supplementary file 1 [file Data_Sheet_1.pdf]

Table 1. Genotypes for patients at baseline (T0).

	NGT	IGT & CFRD
Baseline (T0)		
Patients, total (*n*)	58	24
Patients, with severe mutation* (*n*)	31	21
Patients, with severe mutation* (%)	53%	88%
ΔF508 Homozygous (*n*)	18	11
ΔF508 Heterozygous (*n*)	35	11
Other alleles (*n*)	Unknown (2), 2184delA (1), 2789+2insA (1), 2789+5G->A (3), 3272-26A->G (2), 574delA (1), 5T (1), 621+1G->T (2), A455E (1), D1152H (2), E585X (1), G542X (1), G551D (4), P67L (5), R117H (3), R334W (3), R347H (1), W1282X (1)	Unknown (2), 621+1G->T (1), 1717-1G->A (1), 1898+1G->A (1), 711+1G->T (1), E60X (1), N1303K (1), Q220X (1), Q493X (1), R506T (1)
No ΔF508 (*n*)	5	2
Genotypes	18G->T/1-8G->C<br>711+1G->T/2789+5G->A<br>G551D/1717-1G->A<br>N1303K/-<br>V520F/1078delT	G542X/-<br>V520F/1078delT

*Severe mutation defined as Class I/II, with genotypes classified using CFTR2 database. CFRD: cystic fibrosis related diabetes, IGT: impaired glucose tolerance, NGT: normal glucose tolerance, T0: Baseline.

Table 2. Genotypes for patients at follow-up (T1).

	NGT	IGT & CFRD

One-year Follow Up (T1)		
Patients, total (*n*)	41	13
Patients with severe mutation* (*n*)	25	12
Patients with severe mutation* (%)	61%	92%
ΔF508 Homozygous (*n*)	17	7
ΔF508 Heterozygous (*n*)	19	5
Other alleles (*n*)	Unknown (1), 2789+2insA (1), 2789+5G->A (2), 3272-26A->G (1), 574delA (1), 621+1G->T (1), G542X (1), G551D (2), P67L (3), R117H (2), R334W (2), R347H (1), W1282X (1)	Unknown (1), 621+1G->T (1), 711+1G->T (1), Q220X (1), R506T (1)
No ΔF508 (*n*)	5	1
Genotypes	18G->T/1-8G->C<br>711+1G->T/2789+5G->A<br>G551D/1717-1G->A<br>N1303K/-<br>V520F/1078delT	V520F/1078delT

*Severe mutation defined as Class I/II, with genotypes classified using CFTR2 database. CFRD: cystic fibrosis related diabetes, IGT: impaired glucose tolerance, NGT: normal glucose tolerance, T1: 1 year follow-up.

Table 3. Differences between timepoints by number of patients with severe genotype.

	**NGT**	**IGT & CFRD**
Change		
T0-T1 difference, total (*n*)	17	11

T0-T1 difference, patients with severe mutation* (*n*)	6	9
T0-T1 difference, patients with severe mutation* (%)	35%	82%

*Severe mutation defined as Class I/II, with genotypes classified using CFTR2 database. CFRD: cystic fibrosis related diabetes, IGT: impaired glucose tolerance, NGT: normal glucose tolerance, T0: Baseline, T1: 1 year follow-up.

Table 4. Independent Samples T-Test for Antibiotic Days per group

**Group**	***n***	**Mean Days**	**Standard Deviation**
NGT	35	5	10
IGT/CFRD	11	25	25
		**Mean Difference**	**20 Days**
		***P*-Value**	**0.026**

CFRD: cystic fibrosis related diabetes, IGT: impaired glucose tolerance, NGT: normal glucose tolerance.
